# Solitary Fibrous Tumors of the Pleura With Extensive Intrathoracic Occupation: Report of Two Cases

**DOI:** 10.1155/crpu/8899120

**Published:** 2026-08-28

**Authors:** Renzo Villanueva-Villegas, Cristian Morán-Mariños, Ruth Huaman-Achahui, Claudia Melendez-Davila, Briam Benito-Condor, José Monroy-Palomino

**Affiliations:** ^1^ Department of Pulmonology, Hospital Nacional Dos de Mayo, Lima, Peru; ^2^ Bibliometrics Research Unit, Vice-Rectorate for Research, Universidad San Ignacio de Loyola, Lima, Peru, usil.edu.pe; ^3^ Department of Thoracic and Cardiovascular Surgery, Hospital Nacional Dos de Mayo, Lima, Peru

**Keywords:** pleura, solitary fibrous tumor, thoracic neoplasms, thoracotomy, tumor recurrence

## Abstract

Solitary fibrous tumors of the pleura (SFTPs) are rare mesenchymal neoplasms that typically originate from the visceral pleura. We report two cases of giant pleural SFTs involving the left hemithorax. The first case involved a 59‐year‐old woman presenting with two synchronous tumors, whereas the second case concerned a 39‐year‐old man with a single large intrathoracic mass. The coexistence of two distinct SFTs within the same hemithorax, as observed in the first patient, is exceptionally rare and may suggest a multicentric pleural origin or intrapleural dissemination. Both patients presented with progressive respiratory symptoms and mediastinal shift on chest computed tomography. Histopathological diagnosis was confirmed by percutaneous biopsy and immunohistochemical positivity for CD34, CD99, and BCL2. Complete tumor resection was achieved through posterolateral thoracotomy in both cases. One patient developed early postoperative recurrence during follow‐up. These cases highlight the clinical heterogeneity of pleural SFTs and emphasize the diagnostic and surgical challenges posed by giant intrathoracic tumors. Accurate histopathological confirmation and complete surgical resection remain essential for optimal management, whereas long‐term surveillance is warranted due to the potential risk of recurrence.

## 1. Introduction

Solitary fibrous tumors of the pleura (SFTPs) are rare mesenchymal neoplasms with an estimated incidence of approximately 2.8 per 100,000 individuals and account for less than 5% of all pleural tumors [1, 2]. These tumors arise from dendritic mesenchymal cells distributed across multiple anatomical sites. Although SFTs may occur in locations such as the mediastinum, orbit, or central nervous system, the pleura represents the most common site of origin [3].

At the molecular level, SFTs are characterized by the NAB2–STAT6 gene fusion, which plays a central role in tumorigenesis and biological behavior [4]. Despite advances in molecular characterization, no specific environmental risk factors including tobacco use or asbestos exposure have been definitively associated with their development [5]. Most tumors arise from the visceral pleura, typically within the lower hemithorax, accounting for approximately 80% of reported cases [5, 6]. Due to their slow and indolent growth, early clinical manifestations are often absent. As the tumor enlarges, patients may develop symptoms such as cough, chest pain, dyspnea, or thoracic discomfort. Lesions are generally classified as giant SFTs when they exceed 20 cm in diameter, frequently causing compression and displacement of adjacent thoracic structures and leading to more pronounced clinical manifestations [6, 7].

Complete surgical resection remains the cornerstone of treatment, although local recurrence has been reported in approximately 8% of cases, underscoring the importance of long‐term surveillance [8, 9]. Despite advances in diagnostic imaging and surgical management, giant pleural SFTs remain uncommon and may present significant diagnostic and therapeutic challenges due to their size, mass effect, and variable biological behavior.

In this context, detailed clinical descriptions of unusual presentations remain valuable to improve understanding of the disease spectrum. Here, we report two cases of slowly progressive giant pleural SFTs, including an exceptional presentation with two synchronous tumors arising within the same hemithorax. Both cases are presented in accordance with the CARE case report guidelines [10].

## 2. Case Presentations

### 2.1. Case 1

A 59‐year‐old woman from the Peruvian Amazon, with arterial hypertension and 20 years of biomass smoke exposure, presented with a two‐year history of progressive left‐sided chest pain, dyspnea (mMRC Grade 3), and 10‐kg weight loss. She sought emergency care due to symptom worsening. On admission, she was hemodynamically stable (BP 128/68 mmHg, HR 98 bpm, RR 28 bpm, SpO_2_ 91%). Physical examination revealed reduced chest expansion, absent breath sounds, and dullness to percussion over the left hemithorax.

**Figure 1:**
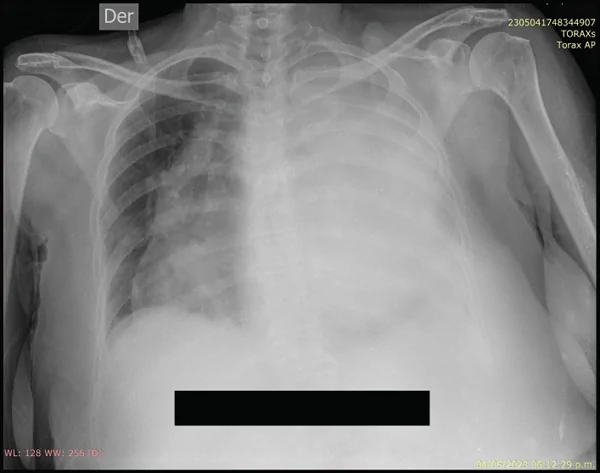
Chest radiograph demonstrating total opacification of the left hemithorax with mediastinal displacement.

**Figure 2:**
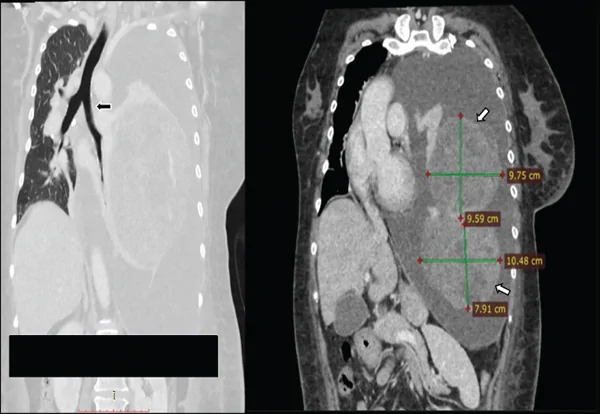
Coronal contrast‐enhanced chest CT demonstrating two giant pleural‐based masses with heterogeneous density and internal calcifications, causing extensive occupation of the left hemithorax and compression of adjacent thoracic structures. White arrows indicate the two masses, whereas the black arrow shows contralateral mediastinal and tracheal displacement. Maximum dimensions are indicated.

Chest radiography demonstrated a mass occupying approximately 90% of the left hemithorax with contralateral mediastinal and tracheal deviation (Figure 1). Contrast‐enhanced CT revealed two well‐defined, partially enhancing encapsulated masses with homogeneous density (Figure 2).

Image‐guided biopsy revealed a spindle‐cell mesenchymal neoplasm composed of relatively uniform oval‐to‐spindle cells arranged in a patternless architecture within a collagen‐rich stroma. No overt high‐grade cytological atypia was identified in the limited biopsy sample. Immunohistochemistry showed tumor‐cell positivity for CD34, CD99, and BCL2, with a Ki‐67 proliferation index of 3%. Nuclear STAT6 immunohistochemistry could not be performed because the antibody was unavailable at our institution at the time of diagnosis.

**Figure 3:**
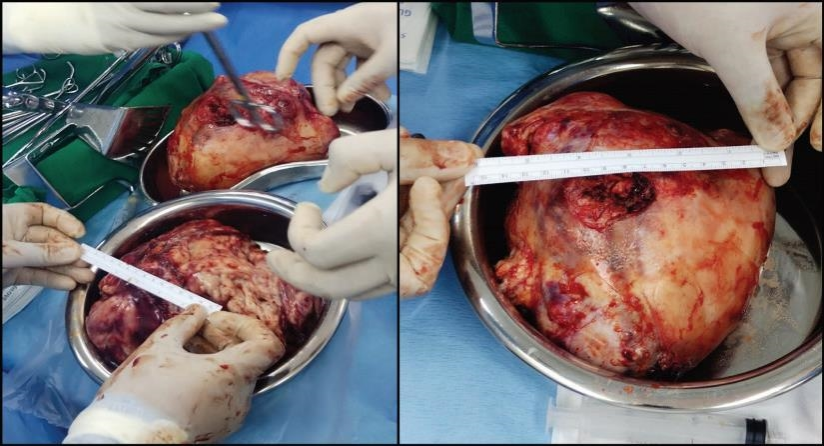
Resected surgical specimens of two pleural solitary fibrous tumors, showing macroscopic encapsulation.

**Figure 4:**
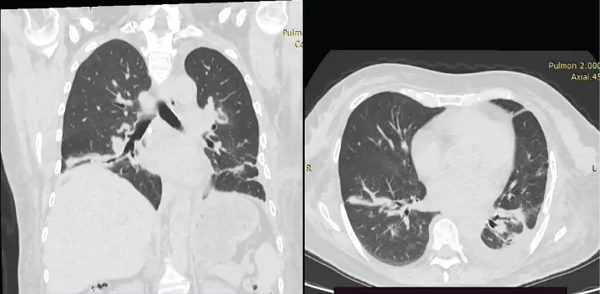
Postoperative chest CT scan showing no residual pleural masses after complete tumor resection.

Left posterolateral thoracotomy identified two solid tumors: one encapsulated mass (15 × 16 × 10 cm; 1299 g) arising from the visceral pleura, and a second nonencapsulated lesion (20 × 16 × 10 cm) firmly adherent to the diaphragm (Figure 3). Macroscopically complete resection was achieved without intraoperative complications. Postoperative recovery was uneventful, with full radiologic lung re‐expansion (Figure 4), and the patient was discharged in good clinical condition.

Histopathological examination of the two resected specimens showed concordant morphological features and confirmed the diagnosis of SFTPs. Both lesions were composed of bland oval‐to‐spindle cells arranged in a patternless growth pattern within a collagen‐rich stroma. Variable cellularity and branching, thin‐walled hemangiopericytoma‐like vessels were observed. The tumor–host interface was predominantly circumscribed. No tumor necrosis, marked nuclear pleomorphism, or dedifferentiated component was identified.

A quantitative mitotic count was not documented in the original pathology report. Although both tumors were macroscopically removed, microscopic surgical margin status was also not reported.

**Figure 5:**
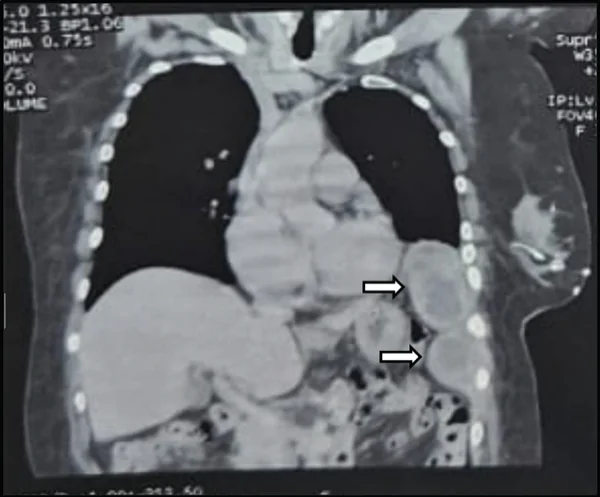
Follow‐up chest CT scan showing biopsy‐confirmed local recurrence in the left hemithorax near the previous surgical field.

At 6 months, surveillance chest CT detected a new ipsilateral lesion involving segments S4 and S5 of the left lung, located adjacent to the previous surgical field, which was interpreted as local recurrence rather than distant disease (Figure 5). A repeat image‐guided biopsy was performed, showing histopathological features consistent with recurrent solitary fibrous tumor of the pleura. Because the patient lived in the Peruvian Amazon and had returned to her place of origin after surgery, further management was coordinated with her local healthcare network, and she remained under clinical and radiological follow‐up. No additional treatment was administered at our institution during the available follow‐up period.

### 2.2. Case 2

A 39‐year‐old man from Lima, Peru, with no relevant medical history, presented with a 15‐month history of progressive dyspnea on moderate exertion, left‐sided chest pain, and unquantified weight loss. He sought emergency care due to worsening respiratory symptoms. On admission, he was hemodynamically stable, with blood pressure of 132/70 mmHg, heart rate of 102 bpm, respiratory rate of 28 breaths per minute, and oxygen saturation of 93% on room air.

**Figure 6:**
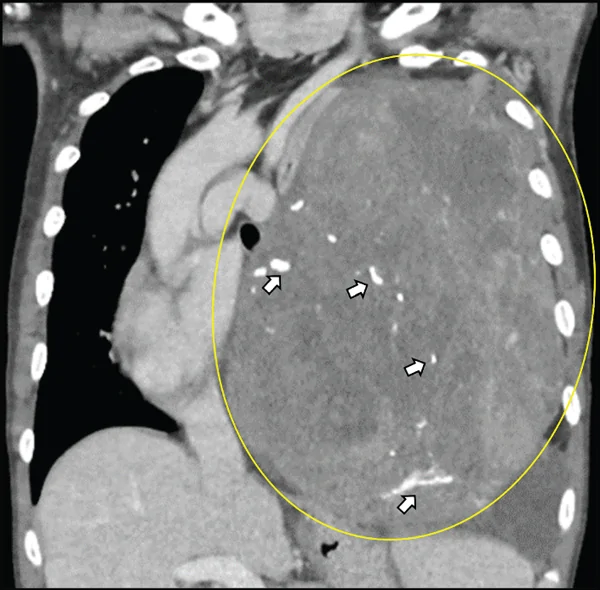
Preoperative coronal chest CT demonstrating a giant heterogeneous pleural‐based mass occupying most of the left hemithorax and causing marked compression of adjacent thoracic structures. The yellow outline delineates the tumor margins, and white arrows highlight internal calcifications.

Chest computed tomography revealed a giant mass occupying nearly the entire left hemithorax, producing mediastinal shift, heterogeneous contrast enhancement, and multiple internal calcifications (Figure 6). Image‐guided percutaneous biopsy suggested a low‐grade mesenchymal tumor.

**Figure 7:**
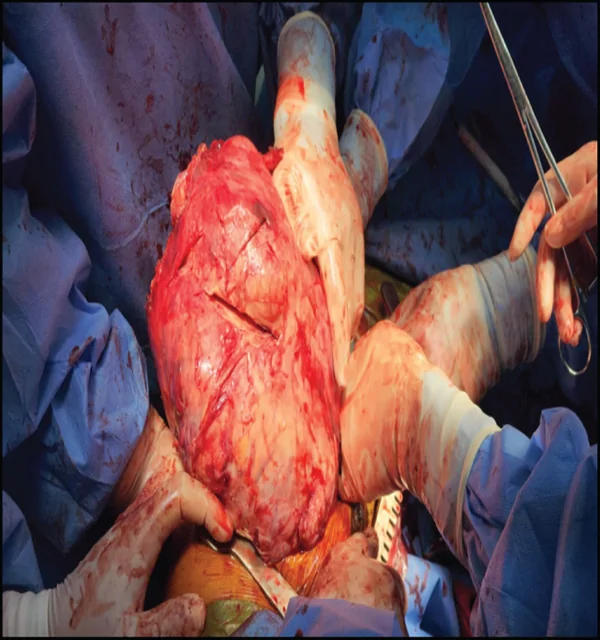
Intraoperative image showing en bloc resection of a giant pleural solitary fibrous tumor.

Surgical resection was performed through a left posterolateral thoracotomy under general anesthesia with double‐lumen endotracheal intubation. Intraoperatively, a large, predominantly encapsulated mass measuring 14 × 18 × 24 cm with fibrous consistency was identified arising from the visceral pleura and occupying most of the left hemithorax (Figure 7). The tumor showed loose adhesions to the pericardium and diaphragm, with focal infiltration of the parietal pleura, costophrenic angle, and adjacent ribs.

An en bloc resection was performed, including partial pleural decortication and segmental resection of the involved ribs. Macroscopically complete tumor removal was achieved without major intraoperative complications.

Histopathological examination of the resected specimen confirmed a solitary fibrous tumor of the pleura. Microscopically, the lesion was composed of bland oval‐to‐spindle cells arranged in a patternless growth pattern, with alternating areas of cellularity within a collagen‐rich stroma. Immunohistochemistry demonstrated tumor‐cell positivity for CD34, CD99, and BCL2. The Ki‐67 labeling index was approximately 5%. Nuclear STAT6 immunohistochemistry could not be performed because the antibody was unavailable at our institution during the diagnostic work‐up. A quantitative mitotic count was not documented in the original pathology report. The report also did not provide a quantitative assessment of tumor necrosis or formal microscopic surgical margin status.

**Figure 8:**
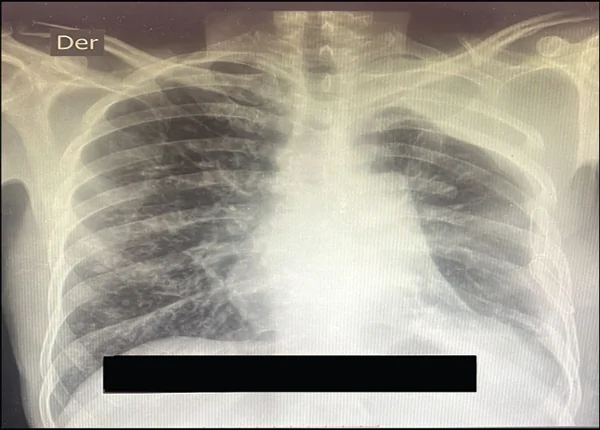
Postoperative chest radiograph demonstrating lung re‐expansion after resection of a giant pleural solitary fibrous tumor.

The immediate postoperative course was favorable, without documented major in‐hospital complications, and follow‐up imaging before discharge showed adequate pulmonary re‐expansion (Figure 8). Three months after discharge, the patient was admitted to another hospital with severe pneumonia and subsequently died. Because the terminal event occurred outside our institution, we were unable to access the complete medical records, microbiological results, or follow‐up chest imaging from that admission. Therefore, a concomitant tumor recurrence, delayed postoperative complication, or other contributing condition could not be definitively assessed.

## 3. Discussion

SFTPs are rare mesenchymal neoplasms of fibroblastic origin, first described by Klemperer and Rabin in 1931, and classically associated with the visceral pleura [11]. Although they account for less than 5% of all pleural tumors, their clinical spectrum is broad, ranging from small, incidentally detected lesions to giant masses that cause severe compression of the lung, mediastinum, diaphragm, and adjacent thoracic structures [12].

In the present report, both patients had giant left‐sided pleural tumors with massive intrathoracic occupation. The largest lesions measured 20 cm in Case 1 and 24 cm in Case 2. Both patients presented with progressive dyspnea, chest pain, and weight loss, symptoms that are usually related to mass effect in large SFTPs. In contrast, smaller tumors are frequently asymptomatic and may be detected incidentally during imaging performed for unrelated reasons [13]. No episodes of hypoglycemia or other paraneoplastic manifestations suggestive of Doege–Potter syndrome were documented in either patient.

Preoperative diagnosis remains challenging. Chest CT typically demonstrates well‐circumscribed pleural‐based masses with homogeneous or heterogeneous contrast enhancement and may show mediastinal displacement, lung compression, or contact with the diaphragm and chest wall [14]. However, imaging findings alone cannot reliably distinguish benign from malignant SFTP, nor can they always differentiate multifocal disease from recurrence or pleural dissemination. In our cases, imaging demonstrated large masses occupying most of the left hemithorax, and the diagnosis was supported by percutaneous biopsy and subsequent histopathological evaluation of the resected specimens.

Definitive diagnosis requires integration of morphology, immunohistochemistry, and clinicopathological risk assessment. Microscopically, SFTPs are usually composed of spindle‐shaped cells arranged in a patternless architecture within a collagen‐rich stroma, often with branching hemangiopericytoma‐like vessels. Immunohistochemical staining commonly demonstrates positivity for CD34, BCL2, and CD99, whereas nuclear STAT6 expression is currently considered the most useful diagnostic marker because it serves as a surrogate for the NAB2–STAT6 gene fusion, a molecular hallmark of SFTs [15]. In our series, both tumors showed immunoreactivity for CD34, CD99, and BCL2, with low proliferative activity based on Ki‐67 indices of 3% and 5%. STAT6 immunohistochemistry could not be performed because the antibody was not available at our institution during the diagnostic work‐up, which represents a diagnostic limitation.

An unusual feature of Case 1 was the presence of two synchronous tumors within the same hemithorax. Although SFTPs are typically solitary lesions, rare reports of multiple unilateral tumors have been described [16]. Taştepe et al. reported two synchronous benign localized fibrous tumors arising from the visceral pleura in the same hemithorax, and Watanabe et al. recently described multiple unilateral and multicentric SFTPs, all positive for CD34, CD99, BCL2, and STAT6 [16, 17]. These reports support the concept that multifocal pleural tumorigenesis, although uncommon, can occur.

Our first case shares similarities with these previous reports because both tumors were synchronous and located in the left hemithorax. However, it differs in several clinically relevant aspects: both tumors were giant, one lesion was nonencapsulated and firmly adherent to the diaphragm, and the patient developed an early ipsilateral lesion near the previous surgical field, which was confirmed by repeat biopsy and interpreted as local recurrence. This behavior highlights the difficulty of distinguishing multicentric disease, local recurrence, and pleural dissemination in patients with multiple SFTPs, particularly when tumors are large and anatomically complex.

Although large tumor size has historically been associated with malignancy, size alone does not necessarily predict aggressive biological behavior. Large SFTPs may remain histologically low grade in the absence of increased mitotic activity, necrosis, marked pleomorphism, hypercellularity, vascular invasion, or positive margins [18]. Nevertheless, tumor size greater than 10 cm has been consistently recognized as a risk factor for recurrence and metastasis, and giant tumors should undergo formal risk stratification using established criteria such as England′s criteria and the Demicco model [19–21]. In our cases, low Ki‐67 values suggested low proliferative activity; however, the large tumor size and the early recurrence in Case 1 justify prolonged surveillance.

Complete surgical resection remains the cornerstone of treatment for localized SFTP. Minimally invasive approaches such as video‐assisted thoracoscopic surgery may be appropriate for small or pedunculated lesions, whereas open thoracotomy is generally preferred for giant tumors to ensure adequate exposure, safe vascular control, and complete removal [2, 22]. In our series, both patients underwent left posterolateral thoracotomy with macroscopically complete resection. In Case 2, en bloc resection was required because of focal involvement of the parietal pleura, costophrenic angle, and adjacent ribs. These findings illustrate the surgical complexity of giant SFTPs and the need for individualized operative planning.

Recurrence after resection is uncommon but clinically relevant. Recent systematic reviews estimate an overall postresection recurrence rate of approximately 8%–13% [2]. Importantly, recurrence risk differs substantially according to histological behavior, being lower among benign SFTPs and higher among malignant tumors, with reported rates of approximately 3% and 22%, respectively [8, 23, 24]. Therefore, broad recurrence ranges should be interpreted cautiously, and recurrence risk should be discussed according to tumor biology, surgical margins, mitotic activity, necrosis, Ki‐67 index, and tumor size.

For unresectable, metastatic, or recurrent disease, systemic treatment may be considered. Antiangiogenic agents targeting VEGF‐related pathways, including pazopanib, sunitinib, sorafenib, and bevacizumab‐based regimens, have shown greater activity than conventional chemotherapy in advanced SFT, reflecting the vascular biology of these tumors [22, 25]. However, the role of adjuvant chemotherapy or radiotherapy after complete resection remains uncertain and should be individualized according to recurrence risk, margin status, and multidisciplinary assessment.

Long‐term surveillance is essential because SFTPs may recur even after apparently complete resection and even when initial histology appears low grade. Imaging follow‐up every 6 months during the first 2 years and annually thereafter for up to 10–15 years has been proposed due to the unpredictable biological behavior of these tumors [24, 26, 27]. Our report also has limitations related to incomplete postdischarge follow‐up. In Case 1, local recurrence was confirmed by repeat biopsy, but subsequent management continued outside our institution after the patient returned to her home region in the Peruvian Amazon. In Case 2, death occurred 3 months after discharge during hospitalization for severe pneumonia at another center; however, the absence of complete external records, follow‐up imaging, and autopsy findings prevented us from determining whether recurrence, delayed postoperative complications, or other factors contributed to the fatal outcome.

In conclusion, giant pleural SFTPs are rare tumors that pose important diagnostic, pathological, and surgical challenges. This report describes a rare presentation of synchronous giant SFTPs within the same hemithorax, comparable with previously reported multifocal unilateral cases but distinguished by massive intrathoracic occupation and early biopsy‐confirmed local recurrence. Accurate diagnosis requires integration of histopathological features and immunohistochemical findings, including the typical expression of CD34, CD99, and BCL2, with STAT6 nuclear staining recommended whenever available as a highly specific diagnostic marker. Risk stratification should also consider tumor size, mitotic activity, necrosis, Ki‐67 index, and margin status. Complete surgical resection remains the mainstay of treatment, and prolonged surveillance is essential because recurrence may occur even after apparently curative surgery.

## Funding

No funding was received for this manuscript.

## Conflicts of Interest

The authors declare no conflicts of interest.

## Supporting information


**Supporting Information.** Additional supporting information can be found online in the Supporting Information section.

## Data Availability

Data sharing is not applicable to this article as no datasets were generated or analyzed during the current study.
