# Supplementary material for: Solitary Fibrous Tumors of the Pleura With Extensive Intrathoracic Occupation: Report of Two Cases
Source: Case Rep Pulmonol. 2026 Aug 28;2026:8899120. doi: 10.1155/crpu/8899120 (PMC13523727; doi:10.1155/crpu/8899120)
Supplement: Supplementary file 1 [file CRPU-2026-8899120-s001.pdf]

CARE
case report guidelines

CARE Checklist of information to include when writing a case report

(cc) BY-NC-ND

Topic	Item	Checklist item description	Reported on Line
Title	1	The diagnosis or intervention of primary focus followed by the words "case report"	1
Key Words	2	2 to 5 key words that identify diagnoses or interventions in this case report, including "case report"	46
Abstract (no references)	3a	Introduction: What is unique about this case and what does it add to the scientific literature?	31-36
	3b	Main symptoms and/or important clinical findings	36-37
	3c	The main diagnoses, therapeutic interventions, and outcomes	37–39
	3d	Conclusion—What is the main "take-away" lesson(s) from this case?	41–45
Introduction	4	One or two paragraphs summarizing why this case is unique (**may include references**)	49 - 69
Patient Information	5a	De-identified patient specific information.	77-80 and 96-1000
	5b	Primary concerns and symptoms of the patient.	77-80 and 96-98
	5c	Medical, family, and psycho-social history including relevant genetic information	77-78
	5d	Relevant past interventions with outcomes	N/A
Clinical Findings	6	Describe significant physical examination (PE) and important clinical findings.	80-82
Timeline	7	Historical and current information from this episode of care organized as a timeline	77-94 and 96-118
Diagnostic Assessment	8a	Diagnostic testing (such as PE, laboratory testing, imaging, surveys).	83–87 and 102–105
	8b	Diagnostic challenges (such as access to testing, financial, or cultural)	N/A
	8c	Diagnosis (including other diagnoses considered)	86-87 and 115-116
	8d	Prognosis (such as staging in oncology) where applicable	118
Therapeutic Intervention	9a	Types of therapeutic intervention (such as pharmacologic, surgical, preventive, self-care)	88-91 and 106-113
	9b	Administration of therapeutic intervention (such as dosage, strength, duration)	106–113
	9c	Changes in therapeutic intervention (with rationale)	91–94 and 116-118
Follow-up and Outcomes	10a	Clinician and patient-assessed outcomes (if available)	91–94 and 116-118
	10b	Important follow-up diagnostic and other test results	118
	10c	Intervention adherence and tolerability (How was this assessed?)	N/A
	10d	Adverse and unanticipated events	N/A
Discussion	11a	A scientific discussion of the strengths AND limitations associated with this case report	146–156
	11b	Discussion of the relevant medical literature **with references**.	146–176
	11c	The scientific rationale for any conclusions (including assessment of possible causes)	157–168
	11d	The primary "take-away" lessons of this case report (without references) in a one paragraph conclusion	177–183
Patient Perspective	12	The patient should share their perspective in one to two paragraphs on the treatment(s) they received	N/A
Informed Consent	13	Did the patient give informed consent? Please provide if requested	Yes ☒ No ☐
